# Interplay between Liposomes and IgM: Principles, Challenges, and Opportunities

**DOI:** 10.1002/advs.202301777

**Published:** 2023-05-07

**Authors:** Huan Wang, Shiqi Lin, Xiying Wu, Kuan Jiang, Huiping Lu, Changyou Zhan

**Affiliations:** ^1^ School of Pharmacy Naval Medical University Shanghai 200433 P. R. China; ^2^ Department of Pharmacy, Shanghai Pudong Hospital, Fudan University Pudong Medical Center & Department of Pharmacology School of Basic Medical Sciences Fudan University Shanghai 201399 P. R. China; ^3^ Shanghai Skin Disease Hospital Tongji University School of Medicine Shanghai 200443 China; ^4^ Shanghai Engineering Research Center for Synthetic Immunology Fudan University Shanghai 200032 P. R. China

**Keywords:** complement activation, delivery process, immunoglobulin M, liposomes

## Abstract

Liposomes have received tremendous attention as a class of versatile pharmaceutical vehicles of great potential over the past several decades. However, the application of liposomes encounters major challenges due to the knowledge gaps in their in vivo delivery process. Immunoglobulin M (IgM) displays both pervasiveness and complexity in regulating the biological functions as well as eliciting adverse effects of liposomes. Understanding, mitigating, and exploiting the duality of IgM are prerequisites for achieving various biomedical applications of liposomes. In this review, the intricate relationship between liposomes and their biological environments has been summarized, with an emphasis on the regulatory effects of IgM on in vivo performance of liposomes. Corresponding solutions have also been discussed to evade IgM‐mediated opsonization for safe and efficient drug delivery.

## Graphical abstract

In this review, the intricate relationships between the regulatory effects of immunoglobulin M (IgM) and liposome performance in vivo are organized and contemplated. Corresponding strategies are proposed to evade IgM‐mediated opsonization for safe and efficient drug delivery. With this mechanistic knowledge as guidance, it is beneficial to design liposomes for in vivo delivery in a controlled manner.

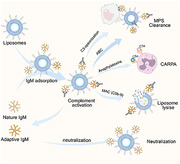

## 1 Introduction

The use of liposomes as potential drug delivery systems was envisioned shortly after their discovery by Bangham in 1964.^[^ ^1^ ^]^ After years of extensive studies, several major advances have been made in liposomal research, including the development of immunoliposomes to achieve targeted delivery, stealth liposomes to prolong circulation time, and stimuli‐responsive liposomes to release encapsulated drugs under specific triggers at the desired sites.^[^ ^2^, ^3^, ^4^ ^]^ Meanwhile, some liposomal products, with the example of Doxil, DaunoXome, Myocet for the treatment of cancers and Ambisome for antifungal therapy, have reached the market.^[^ ^5^, ^6^, ^7^ ^]^ Despite these successes, substantial challenges remain for clinical translation and application of liposomes. For example, the delivery efficiency of liposomes was only 0.5% as reported, representing nonspecific interaction rather than specific targeting.^[^ ^8^ ^]^ Increasing adverse effects represented by hypersensitivity reactions make the clinical application of liposomal therapeutics now in a plight.^[^ ^9^ ^]^ In addition, the major improvement of Doxil appears to be the reduction of cardiotoxicity compared to free doxorubicin, rather than a significant amelioration of the survival time of patients. Significant improvements are still required to promote the safety and efficiency of delivering therapeutics to target cells.

This gap between laboratory findings and clinical application largely results from the limited knowledge of the in vivo delivery process of liposomes. The mechanistic study on reciprocal interaction between liposomes and the immune system is a key scientific issue in the field of nano‐biological effects and for rational design of liposome‐based nanomedicines. In complex pathophysiological environments, liposomes interact with numerous plasma proteins to form the “protein corona” that regulates circulation time, biodistribution, drug release, immunotoxicity, and cellular interactions of liposomes.^[^ ^10^, ^11^ ^]^ Validating the key plasma proteins that dominate in vivo performance of liposomes and revealing the regulatory mechanisms are vital for guiding rational medication in clinical practice. Among the numerous plasma proteins, immunoglobulin M (IgM) has received increasing attention due to its ubiquity and complexity in regulating the biological functions of liposomes in vivo.

In this review, we first describe ubiquitous IgM adsorption on liposomal surfaces. Then, modulatory effects of IgM on the in vivo performance of liposomes (PEGylated small unilamellar vesicle with a diameter around 100 nm) have been highlighted, such as altered pharmacokinetics, reduced pharmacodynamics, and induction of adverse reactions. We also summarize the corresponding strategies to improve in vivo performance of liposomes by manipulating the reciprocal interaction between IgM and liposomes.

## 2 Key Plasma Protein in Liposomal Protein Coronas

### 2.1 Inevitable Formation of Protein Coronas

**Figure 1:**
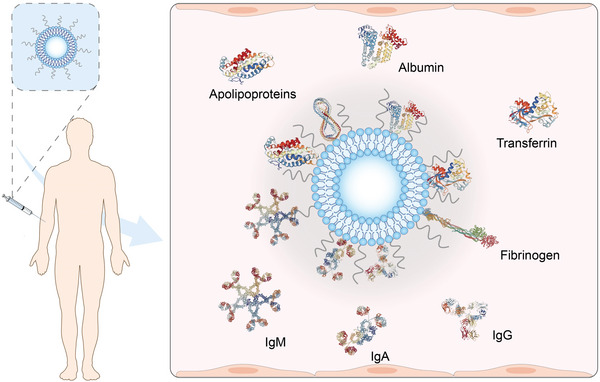
Inevitable formation of protein coronas on the liposomal surface during circulation. Once administered intravenously, liposomes are immediately covered by various proteins including albumin, immunoglobin, apolipoprotein, and fibrinogen, forming the so‐called “protein coronas.”

The knowledge gap in the interplay between liposomes and the physiological environment, for example, the blood and interstitial fluids, is probably one of the major reasons responsible for the limited clinical use of liposome‐based therapeutics. Most of the commercially available liposomal products are administered systemically. Once administered, liposomes are immediately surrounded by various plasma proteins (e.g., albumin, immunoglobin, apolipoprotein, and fibrinogen), forming the so‐called “protein coronas” as shown in **Figure** **1**.^[^ ^12^, ^13^ ^]^ The protein coronas usually consist of two layers, the inner layer (hard corona) formed by proteins tightly bound to the liposome and the outer layer (soft corona) composed of loosely bound proteins. The boundary between the two layers is blurred and the corona is always in a thermodynamic equilibrium during blood circulation.

There are dozens of plasma proteins with varying identities and quantities, and the composition of protein coronas can be greatly influenced by the complexity of the physiological environment. As a result, the impact of protein adsorption on liposomes can be multifold. It might mask the pre‐set chemical or biological functions of liposomes and govern the in vivo fate, including circulation lifetime, biodistribution, cellular uptake, and targeting ability. Even though the liposomes are surface‐modified for certain chemical or biological functions, they can be easily masked by the corona. Moreover, the hydration layer around the liposomes is displaced upon protein binding, and the resulting protein aggregation and conformational changes may trigger immune responses in hosts.^[^ ^11^, ^14^ ^]^

### 2.2 Ubiquitous IgM Adsorption

Hundreds of plasma proteins have been confirmed to deposit on liposomal surfaces, some of which might have major impacts on the in vivo fates of liposomes, while others do not.^[^ ^15^ ^]^ Therefore, validating the key plasma protein(s) dominating the in vivo performance of liposomes and revealing the regulatory mechanisms play crucial roles to guide the precise medication of liposome‐based therapeutics. Situations can be even more complicated because the composition and content of plasma proteins undergo dramatic changes in patients during disease progression, which severely exacerbates the unpredictability of the characteristics of protein coronas. Kostarelos et al. described the formation of human‐derived protein coronas on Caelyx (doxorubicin‐encapsulated PEGylated liposomes) following the collection of liposomes from the blood circulation of ovarian cancer patients. Immunoglobulins, lipoproteins, and complement proteins were found to be the most abundant proteins, accounting for 28%, 9%, and 4% of the total protein content, respectively.^[^ ^16^ ^]^ Among immunoglobulins, IgM (Ighm, lg mu chain C region) was found to be the most abundant in liposomal protein corona, whose content was even higher than that of IgG (Ighg1 or Ighg2b, Ig gamma‐l or 2B chain C region).^[^ ^17^, ^18^, ^19^ ^]^

Although surface modification endows liposomes with better in vivo properties such as prolonged circulation time or specific targeting ability, it may also change the corona composition. By preparing liposomes with different targeting ligands (e.g., peptides and small molecules) and molar ratios, we identified that IgM was ubiquitously adsorbed on liposomes with different modifications. IgM plays a critical role in complement activation, negatively correlating with the in vivo performance of liposomes,^[^ ^20^ ^]^ which would be discussed in a later section.

In addition, most of the clinically approved liposomal products are PEGylated that are able to elicit anti‐PEG antibodies.^[^ ^21^ ^]^ An important feature of anti‐PEG antibodies is that they can be detected even in “treatment‐naïve” individuals, with a prevalence of 0.2% in 1984, 25% in 2012, 40% in 2016, and a surprising 97.5% in 2019.^[^ ^22^, ^23^, ^24^ ^]^ This phenomenon is commonly known as pre‐existing anti‐PEG antibodies. Antibody isotype analysis showed that pre‐existing anti‐PEG antibodies were 25% IgM, 18% IgG, and 30% both IgG and IgM in the general population.^[^ ^25^ ^]^ A genome‐wide association study in 177 Han Chinese individuals identified the variable segment of immunoglobulin heavy chain locus is associated with IgM antibody responses to PEG, providing novel genetic markers for predicting the risk of allergic reactions and the effectiveness of PEGylated drugs.^[^ ^26^ ^]^ In nearly all animal studies, major serum proteins binding to PEGylated liposomes was identified to be IgM isotype.^[^ ^27^, ^28^ ^]^ The reduced efficacy of PEGylated therapeutics in clinical trials has been reported to be associated with the presence of pre‐existing anti‐PEG antibodies. They dramatically altered the half‐life and therapeutic effect of PEGylated drugs in patients.^[^ ^29^, ^30^ ^]^

### 2.3 Structural and Functional Characteristics of IgM

**Figure 2:**
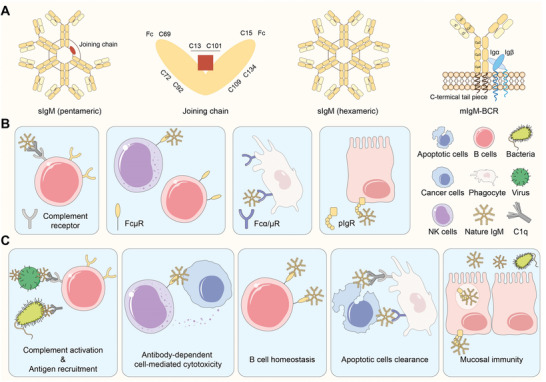
Schematic depiction of IgM structure and biological functions. A) Schematic drawings of sIgM pentamer, joining (J) chain, sIgM hexamer, and mIgM‐BCR complex. B) Some key partners of IgM include complement component C1q and Fc receptors (FcµR, Fc*α*/µR, and pIgR). C) Representative biological functions of sIgM such as pathogens neutralization, classical complement activation associated with C1q, antibody‐dependent cell‐mediated cytotoxicity, B cell hemostasis through FcµR, apoptotic cells clearance mediated by C1q and Fc*α*/µR and transcytosis of sIgM to regulate mucosal immunity.

IgM can be generally divided into two types, monomeric membrane‐bound IgM (mIgM) and secreted IgM (sIgM). mIgM presented on B cellular surface is a main constituent component of B cell receptor (BCR). It controls B cell maturation, activation, and differentiation. sIgM is mainly found in blood, contributing to immune supervision by recognizing pathogens and mediating immune reactions.^[^ ^31^, ^32^ ^]^ sIgM is usually pentameric, containing five pairs of antigen‐binding sites and a joining (J) chain. It is occasionally found in hexamer, with six pairs of antigen‐binding sites but no J chain (**Figure** **2**A). The multimeric structure endows sIgM with potent binding ability, thus sIgM plays a predominant role in affecting the in vivo performance of the administered liposomes compared with other immunoglobins, such as IgG and IgA.

sIgM can be further classified as pre‐immune “natural” IgM (nIgM) and antigen‐induced “adaptive” IgM. nIgM is produced spontaneously by B lymphocytes, mainly B1 cells and marginal zone B (MZB) cells, without specific immunization.^[^ ^33^, ^34^ ^]^ Since it is the first line of defense against pathogens before the formation of germinal centers where adaptive antibodies are formed, nIgM is polyreactive. Adaptive IgM is secreted mainly by B2 cells several days following pathogens exposure. This process is T‐cell dependent and requires interactions between antigens and BCR on B2 cells.^[^ ^35^ ^]^ Adaptive IgM is antigen‐specific, with only limited cross‐reactivity with other antigens. Anti‐PEG IgM falls into this category.

sIgM‐antigen complexes mediated biological activities require interactions of IgM Fc segment with effector molecules including Fc receptors (FcRs, e.g., FcµR, Fc*α*/µR, and pIgR) expressed on various cell membranes and soluble molecules such as complement components (Figure 2B,C). Upon binding with complement components, IgM can activate the complement system with high efficiency. In comparison with IgM pentamer, highly cytolytic IgM hexamer plays a more crucial role in initiating the complement cascade.^[^ ^36^, ^37^ ^]^ In the classical complement pathway, sIgM binds complement component C1q with its central protruding part.^[^ ^38^, ^39^ ^]^ The binding of C1q to sIgM‐antigen complexes activates the C1q‐associated zymogens C1r and C1s. Subsequently, the activated C1 protease cleaves the fourth complement component (C4) into C4b, thereby promoting the classical pathway of complement activation. This unique structure may well explain its 1000‐fold higher C1q binding affinity compared with that of IgG. sIgM has very potent complement‐dependent cytotoxicity (CDC) effects because it is better at fixing complement compared with IgG, which utilized natural killer cells to exert antibody‐dependent cell‐mediated cytotoxicity (ADCC) via Fc gamma receptors.^[^ ^40^, ^41^ ^]^ However, complement activation is a “double‐edged sword”. Unwanted complement activations also cause tissue damage and opsonization of therapeutics. Specifically, complement activation gives rise to powerful anaphylatoxins such as C4a, C3a, and C5a, leading to inflammatory responses and even anaphylaxis in some individuals. The subsequently generated membrane attack complex (C5b‐9) is also responsible for the nonlytic stimulation of vascular endothelial cells, endothelial regulation of hemostasis, and inflammatory cell recruitment.

## 3 IgM Dominating In Vivo Performance of Liposomes

As mentioned before, sIgM ubiquitously deposits on liposomes. Since it plays a vital role in various immune responses including pathogen neutralization, complement activation, apoptotic cell phagocytosis, antigens recruitment, B cell homeostasis, and initiation of adaptive immunity, a better understanding of how liposomes are influenced by IgM‐specific immunity is urgently needed. In most cases, liposomes are naturally recognized by IgM after entry into the bloodstream as potential pathogens so as to trigger processes that protect the host against invaders. Consequently, the therapeutic efficacy of liposome‐based nanomedicines can be reduced by IgM either through altering the pharmacokinetics such as accelerating the blood clearance (ABC) phenomenon, or directly neutralization. Additionally, adverse reactions represented by hypersensitivity reactions (HSRs) caused by PEGylated liposomes have become new issues. In this section, the effect of IgM on the physicochemical properties and in vivo biological performance of liposomes is presented and discussed in depth.

### 3.1 Pharmacokinetics

#### 3.1.1 IgM‐Mediated Complement Activation and Opsonization

Substantial experimental evidence has indicated that nanoparticle‐corona complexes activate the complement system either via the classical, lectin, or alternative pathway. For instance, Chen et al. reported that superparamagnetic iron oxide nanoworms incubated in human plasma and serum rapidly activated the complement system via the alternative pathway.^[^ ^42^ ^]^ Later, they demonstrated that the binding of only a few surface‐bound immunoglobulin molecules, specifically IgG, was needed to trigger complement activation.^[^ ^43^ ^]^ However, the formation of protein coronas is largely affected by the properties of nanomaterials, such as material composition, surface charge, diameter, hydrophobicity, morphology, and stiffness. As for liposomes, Guan et al. verified that nIgM adsorption was more ubiquitous compared with that of IgG on the liposomal surface of ^D^CDX‐modified liposomes and plays important roles in activating complement via the classical pathway.^[^ ^20^ ^]^ Furthermore, anti‐PEG IgM antibodies induced by PEGylated liposomes also activate the complement system.^[^ ^9^, ^44^ ^]^

The primary outcome of liposomes/IgM complex‐mediated complement activation is surface opsonization. Opsonization is an immune process that uses blood components called “opsonins,” such as antibodies and complement proteins, to label non‐self particles for clearance from the bloodstream by phagocytes. Compared to the opsonic effect of IgG (mediated by the interaction of Fc region with the Fc*γ* receptors on macrophages), IgM mediates microbial opsonization through classical complement activation pathway, followed by C3 deposition on the microbial surface with consequent recognition through complement receptor 3 (CR3). CR3 is an opsonic receptor that recognizes C3 fragments such as C3b and iC3b, which binds to multiple sites on the surface of liposomes, aiding recognition and rapid clearance of liposomes by macrophages resident in the reticuloendothelial system bearing complement receptors (e.g., hepatic Kupffer cells, splenic marginal zone and red‐pulp macrophages, blood monocytes, etc). Taking ganglioside‐containing liposomes as an example, naturally occurring low‐affinity anti‐GM ganglioside IgM antibodies (mainly anti‐GM1) are part of the normal antibody repertoire of healthy humans.^[^ ^45^ ^]^ However, in the plasma of patients with autoimmune neuropathies like Guillain–Barré Syndrome, elevated titers of high‐affinity anti‐GM IgM antibodies are detected.^[^ ^46^, ^47^ ^]^ When the GM1‐liposomes are exposed to blood, the complement system is activated through the classical pathway. Subsequently, the activated complement components deposit on the liposomal surface, serving as opsonins to promote the lysis of GM1‐liposomes and the uptake by hepatic Kupffer cells.^[^ ^48^ ^]^

#### 3.1.2 Accelerating Blood Clearance (ABC) Phenomenon

In 2000, Dams et al. discovered the ABC phenomenon in rats, that is, after the first injection of PEGylated liposomes, the repeatedly injected PEGylated liposomes at intervals of several days were rapidly cleared and then accumulated in liver and spleen.^[^ ^49^ ^]^ The ABC phenomenon is ubiquitous in a variety of animals (including rats, mice, rabbits, guinea pigs, mini pigs, and beagle dogs), but with interspecies differences.^[^ ^50^ ^]^ It was reported that the ABC phenomenon plateaued when the interval was 7 days, beyond which it gradually vanished over weeks.^[^ ^9^ ^]^ Notably, the time window to observe the ABC phenomenon is consistent with the circulation of IgM (3 weeks). Many factors affect the strength of the ABC phenomenon such as the dosage of the injected liposomes. For example, Doxil had no ABC phenomenon at a dose of 20 mg m^−2^, while it occurred at a low dose of 0.2 mg m^−2^.^[^ ^50^ ^]^ It was hypothesized that a high dose of PEGylated liposome could induce immune tolerance or B cell anergy in mice.^[^ ^51^ ^]^ Besides PEGylated liposomes, other PEGylated nanocarriers can also have ABC phenomenon, such as PEGylated nanoemulsion,^[^ ^52^ ^]^ PEGylated PLGA nanoparticles,^[^ ^53^ ^]^ PEGylated solid lipid nanoparticles.^[^ ^54^ ^]^

**Figure 3:**
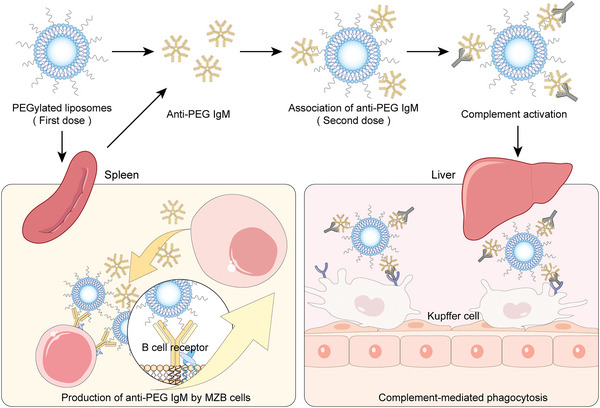
Scheme of the sequential of events from anti‐PEG IgM antibodies productions to accelerated blood clearance of PEGylated liposomes.

Anti‐PEG IgM antibodies elicited following the injection of PEGylated liposomes are shown to account for the ABC phenomenon. The anti‐PEG IgM antibodies exhibit potent neutralizing effects on the subsequently administered PEGylated liposomes in vivo. In animals with circulating anti‐PEG IgM antibodies, the circulation time of PEGylated liposomes is greatly reduced.^[^ ^55^, ^56^ ^]^ Splenic B lymphocytes are involved in recognizing PEGylated liposomes and inducing the production of anti‐PEG IgM antibodies. In rats that received splenectomy before the first injection of PEGylated liposomes, serum IgM concentrations and the amounts of IgM that bound to PEGylated liposomes were significantly decreased and the ABC phenomenon was completely abolished.^[^ ^57^ ^]^ TI immunogenicity (without the involvement of CD4^+^ T cells) is considered to be the main mechanism of antibody induction by PEGylated liposomes due to the typical repeating structure of PEG. In such a process, MZB cells are specialized for the capture of PEGylated liposomes through crosslinking of BCR, and rapidly produce massive but low‐affinity IgM antibodies without the help of T cells within 5 days. Subsequent complement‐mediated opsonization induced by anti‐PEG IgM antibodies is an important part of the ABC phenomenon.^[^ ^58^ ^]^ Therefore, the TI molecular mechanism of ABC could be generalized in the following key steps as shown in **Figure** **3**: administration of initial PEGylated liposomes, recognition of PEGylated liposomes through BCR crosslinking, proliferation and differentiation of MZB cells, formation of anti‐PEG IgM antibodies, deposition of anti‐IgM antibodies on repeated‐administered PEGylated liposomal surface, complement‐mediated opsonization and clearance by cells of the mononuclear phagocyte system (MPS).

Kupffer cells, as the most abundant phagocytic cells in the tissues, are the main contributors to the elimination of PEGylated liposomes and the induction of ABC phenomenon.^[^ ^59^ ^]^ Recently, Deng et al. determined that PEGylated nano‐emulsions (PE) could induce innate immune memory in Kupffer cells. This memory could be transmitted by the PE‐stimulated Kupffer cells to the naïve rats through adoptive transfer, leading to increased hepatic accumulation of PE in the recipient rats, independently of other cells or components until 21 days after the first stimulation.^[^ ^60^ ^]^ The discovery of KC immune memory revisited the relationship between the innate immune system and PEGylated nanomedicine, providing new insights into improving medication in the clinic.

#### 3.1.3 “Off‐Target” Effects

Active targeting strategies that conjugate ligands (e.g., antibodies, peptides, small molecules) to the carrier surface have long been investigated in cancer nanomedicine.^[^ ^61^ ^]^ However, compared to plain liposomes such as Doxil, the number of targeted liposomes that entered the clinical stage is fewer, including MM‐302 (targeting HER2 receptor using an anti‐ErbB2‐scFv for treatment of breast cancer),^[^ ^2^, ^62^ ^]^ 2B3‐101 (targeting glutathione receptor at the blood‐brain barrier for treatment of glioma and brain metastases),^[^ ^63^ ^]^ and MBP‐426 (transferrin‐modified liposomes for gastric, gastroesophageal, or esophageal adenocarcinoma).^[^ ^64^ ^]^ Meanwhile, a growing number of contradictory literature suggests that active targeting strategies do not eventually result in enhanced tumor accumulation.^[^ ^8^ ^]^ The connection between the “off‐target” effect of nanomedicines and the formation of protein coronas has not been observed until recent years.^[^ ^11^, ^65^ ^]^ For instance, Salvati et al. discovered that proteins could hinder the transferrin from binding to both its membrane‐bound receptors and soluble receptors in biological environments. As a result, the targeting molecules on the transferrin‐conjugated nanoparticles were screened and the specificity in targeting was impaired.^[^ ^66^ ^]^ Similarly, protein coronas shields also decreased the binding affinity of anti‐HER2 antibodies,^[^ ^67^ ^]^ cyclic RGD peptides,^[^ ^68^ ^]^ or small molecule ligands^[^ ^69^ ^]^ toward their targets.

Among them, folic acid (FA) is undoubtedly one of the most extensively exploited “star” small molecules to facilitate targeted delivery of nanomedicine by recognition of folate receptors which are overexpressed in many kinds of human cancer cells.^[^ ^70^, ^71^ ^]^ However, all of the FA‐enabled nanomedicine failed in clinical trials (e.g., vintafolide, a folate‐conjugated low‐molecular‐weight chemotherapeutic, clinical trial NCT01170650). Furthermore, FA‐enabled targeted therapies also have inconsistent results in preclinical studies.^[^ ^72^, ^73^ ^]^ Recently, we re‐examined the regulatory roles of the key plasma protein in FA‐modified liposomes (FA‐sLip). It was demonstrated that FA functionalization caused avid deposition of nIgM on the FA‐sLip surface and the binding affinity was positively correlated with the FA modification. The nIgM deposition subsequently activated the complement cascade‐like opsonization by liver and spleen‐resident macrophages, resulting in ABC phenomenon eventually. Most importantly, the binding affinity of FA with folate receptors was inhibited and tumor targeting capacity was severely hindered in folate receptor‐positive tumor models. It is of note that human nIgM also demonstrated strong interaction with FA‐sLip and efficiently activated complement.^[^ ^74^ ^]^ The results have at least three enlightenments for us. First, nIgM was validated as a key plasma protein dominating in vivo performance of FA‐sLip. Second, patient screening was required for the clinical medication of FA‐modified targeted nanomedicines considering the variable titers of IgM in cancer patients. Third, the IgM binding capacity needed to be taken into account in the de novo design of targeted drug delivery systems. Liposomes have long been used as adjuvants to enhance immune effects. FA‐sLip becomes immunogenic just as enhanced immunogenicity of small haptens when combined with a larger carrier. The reason why the enhanced immunogenicity of FA‐enabled nanomedicine was underestimated might be the immune suppression caused by cytotoxic payloads. However, with the continuous emergence of non‐cytotoxic drugs such as antibodies and proteins, the immunogenicity of FA‐sLip as a drug delivery vehicle cannot be ignored.

### 3.2 Reduced Therapeutic Efficacy and Induced Side Effects

#### 3.2.1 IgM Antibodies Neutralization

It is generally accepted that the development of neutralizing antibodies can be expected within days after an initial dose of a biotherapeutic. Neutralizing antibodies, typically low‐affinity IgM‐type immunoglobulins, are initially induced during the initial stages of immune responses. Both TD and TI mechanisms of B‐cell activation have been proposed for IgM generation of immune responses. The early‐onset IgM can later mature into high‐affinity IgG subclasses (IgG1 and IgG4).

In the clinic, neutralizing antibodies produced in patients treated with certain PEGylated biologics are known to be associated with loss of therapeutic efficacy, with examples of PEG‐asparaginase,^[^ ^29^ ^]^ PEG‐uricase,^[^ ^75^, ^76^ ^]^ and PEG‐interferon.^[^ ^30^, ^77^ ^]^ Concerns have been raised regarding the immunogenicity of PEGylated liposomes. Hsieh et al. generated two anti‐PEG antibody mouse models, that is endo *α*PEG (naïve mice administered with PEGylated protein for endogenous anti‐PEG IgM and IgG) and *α*PEG‐PT (naïve mice passively transferred with monoclonal anti‐PEG antibodies). Tumor‐bearing naïve, endo *α*PEG, and *α*PEG‐PT mice were then i.v. treated with ^111^In‐labeled PEGylated doxorubicin‐loaded liposomes. Reduced tumor accumulation and therapeutic efficacy were found in endo *α*PEG and *α*PEG‐PT mice.^[^ ^78^ ^]^ Although direct evidence linking neutralizing antibodies to the limited therapeutic effect of Doxil remains unexplored in clinical trials, it should not be overlooked considering the pre‐existing anti‐PEG IgM antibodies in the population, especially in patients requiring repeated injections.^[^ ^22^, ^24^, ^26^ ^]^

#### 3.2.2 Lysis of Liposomes and Drug Leakage

Once triggered by one of three pathways (classical, alternative, or lectin), complement activation can result in multiple relative functions. For example, C5 along with the other terminal complement factors deposits into the membrane of pathogens to form the membrane attack complex (MAC, C5b‐9). The MAC forms a small channel in the lipid membrane, leading to cellular contents efflux and cell death, which is the main mechanism of complement‐dependent cytotoxicity.^[^ ^79^ ^]^ Pre‐existing or elicited anti‐PEG IgM antibodies by PEGylated liposomes can trigger complement activation. Since the phospholipid composition of liposomes is very similar to that of biological membranes, MAC can also be formed on liposomes. Chen et al. showed that anti‐PEG antibodies (IgM and IgG) bind to PEG molecules on PEGylated liposomal doxorubicin, resulting in complement activation and MAC insertion in the liposomal membrane. Consequently, up to 40% of the encapsulated doxorubicin was released from the liposomes.^[^ ^80^ ^]^ Senti et al. further investigated the outcomes and mechanisms of complement activation following the interaction of anti‐PEG antibodies with different PEGylated lipid‐based nanoparticles, liposomes loaded with different model drugs, and lipid nanoparticles encapsulated with mRNA. They demonstrated that complement activation initiated by anti‐PEG antibodies could potentially compromise the integrity of lipid nanoparticles, leading to unwanted drug release or exposure of mRNA to serum proteins.^[^ ^81^ ^]^ In this case, the protective effect of liposomes was lost, the loaded drug was quickly cleared, and even serious toxic side effects were caused due to the burst release of drugs.

#### 3.2.3 Complement Activation‐Related Pseudo‐Allergy

In recent years, the incidence of side effects of PEGylated liposomes represented by Doxil has increased with their extensive clinical application. Doxil‐related mild to severe hypersensitivity reactions like breathing difficulty, facial swelling, chest pain, flushing, rash, and blood pressure aberrance can occasionally be life‐threatening. The immediate, non‐IgE‐mediated acute hypersensitivity reactions are defined as complement activation‐related pseudo‐allergy (CARPA).

CARPA has been confirmed in both experimental and clinically approved liposomal therapeutics (e.g., Doxil, Ambisome, and DaunoXome), micelles (e.g., Taxol and Taxotere), iron oxides (e.g., Feraheme, Resovist, Feridex) and LNP‐based COVID‐19 mRNA vaccine.^[^ ^82^, ^83^, ^84^, ^85^, ^86^, ^87^ ^]^ For example, up to 45% of patients treated with Doxil without steroids and antihistamines medication developed CARPA, and this reaction still occurred in 4–7.1% of patients premedicated with steroids and antihistamines.^[^ ^88^ ^]^ The phenomenon reflects an unsolved immune dilemma to the clinical application of these nanomedicines, yet the mechanisms are inadequately explored. It is assumed that CARPA of Doxil is caused by PEGylated liposomes as free doxorubicin alone does not induce these hypersensitivity syndromes. In fact, CARPA is closely related to the activation of the complement system. Anaphylatoxins (e.g., C3a and C5a) generated in complement activation specifically bind to their receptors on immune cells (e.g., basophils and mast cells), triggering the release of various vasoactive mediators, including histamine, leukotrienes, tryptase, platelet‐activating factor, thromboxane A2, and prostaglandins. Recent evidence highlighted the causal role of anti‐PEG IgM antibodies in the initiation of CARPA by PEGylated liposomes. Single i.v. injection of a low dose of PEGylated liposomes (Doxebo, without drug compared to Doxil) in pigs induced massive anti‐PEG IgM antibodies formation in blood, with the concentration peaking on days 6–9 and decreasing slowly in 6 weeks. Bolus injections of the same liposomes during seroconversion led to fatal anaphylactic reactions within 2–3 min in immunized pigs. Similar treatments in naïve animals only resulted in minor reactions. The results suggest that the classical complement activation triggered by rapid binding of anti‐PEG IgM antibodies to PEGylated liposomes is closely related to anaphylactoid shock.^[^ ^89^ ^]^ In addition, pre‐existing anti‐PEG antibodies have also been linked to CARPA after the infusion of PEGylated medicines.^[^ ^82^, ^90^ ^]^

## 4 Strategies to Improve In Vivo Performance of Liposomes

### 4.1 Prescreening the Suitable Population for Precise Medication

The IgM‐protein corona varies among individuals due to not only proteome heterogeneity but also disease‐related alterations in IgM titers and structures. For instance, in patients with hyper IgM syndrome, a rare primary immunodeficiency disorder, the blood titers of IgM can be abnormally elevated while the concentrations of IgG, IgA, and IgE are decreased.^[^ ^91^ ^]^ In patients suffering from chronic liver diseases and autoimmune diseases, the IgM is predominantly monomeric instead of multimeric.^[^ ^92^ ^]^ To make matters worse, IgM levels and functions undergo dynamic changes during disease progression and medication, especially in cancer patients receiving chemotherapy, severely exacerbating the unpredictability of liposome clinical use. The immune system disorders in patients may be a crucial factor in individual differences in the efficiency and side effects of clinically approved liposome‐based therapies. It is notable that anti‐PEG IgM antibodies titer in general population also exists individual differences with high antibody levels in a small part of samples.^[^ ^25^ ^]^ Thus, prescreening the suitable population through IgM affinity tests before medication is critical to maximizing the clinical benefits of liposomal therapeutics.

Meanwhile, IgM in pathological conditions can be leveraged to improve liposome‐based therapies. For example, post‐rituximab IgM hypogammaglobulinemia refers to low IgM levels resulting from the administration of rituximab. Rituximab is a CD20‐positive B cell‐targeting chimeric monoclonal antibody. It is currently used to treat lymphoma (e.g., chronic lymphocytic leukemia and non‐Hodgkin lymphoma) and a variety of autoimmune diseases (e.g., systemic lupus erythematosus and rheumatoid arthritis). However, rituximab also kills normal B cell subsets that contributed to IgM production. Clinical data suggest that the prevalence of IgM hypogammaglobulinemia following several cycles of therapy with rituximab ranges between 10% and 58%.^[^ ^93^, ^94^ ^]^ Post‐rituximab IgM hypogammaglobulinemia has caused clinical concerns because of the significant role of IgM in protective immunity, but it provides an ideal time window for the delivery of liposome‐based therapeutics.

### 4.2 Blocking or Depleting the Mononuclear Phagocyte System

The rapid blood clearance of liposomes induced by the MPS significantly restricts the therapeutic efficiency of many liposomal formulations. Administration of macrophage‐depleting toxic compounds, such as gadolinium chloride^[^ ^95^ ^]^ and clodronate liposomes,^[^ ^96^ ^]^ can significantly prolong liposome circulation. However, poisonous compounds accumulated in Kupffer cells will result in cell apoptosis or cell necrosis. It usually takes at least 2 weeks for the recovery of Kupffer cells, during which bacteremia will be fatal to patients. A less aggressive approach is based on the efficient MPS blockade by injecting large doses of foreign substances such as dextran sulfate,^[^ ^97^ ^]^ colloidal carbon,^[^ ^98^ ^]^ liposomes,^[^ ^99^ ^]^ and fat emulsions.^[^ ^100^ ^]^ But dose‐related toxicity considerations limit the application of this strategy in clinical practice and even basic research. An alternative, called MPS‐cytoblockade, is a conceptually different approach by injecting allogeneic anti‐erythrocyte antibodies to force the MPS to boost the clearance of its own intact erythrocytes.^[^ ^101^ ^]^

The hepatic clearance capability, the serum anti‐PEG IgM concentration, and the complement activation intensity are three important contributors to the ABC phenomenon of liposomes. Ishida et al. found that the strength of complement activation in mice increased linearly with the increase of serum anti‐PEG IgM antibody levels. But hepatic clearance of PEGylated liposomes was first increased and then saturated, suggesting that hepatic uptake is a limiting step in the ABC phenomenon.^[^ ^102^ ^]^ However, excessive depletion or blockade of Kupffer cells instead would exacerbate the ABC phenomenon because more circulating liposomes in blood (within a range of concentrations) would stimulate B cells to produce more anti‐PEG IgM antibodies.^[^ ^103^ ^]^

Although liver is the primary organ that contributes to blood clearance of liposomes, the specific mechanisms differ among species. For example, hepatic uptake of liposomes in mice does not involve specific serum opsonin. In contrast, the uptake of liposomes by rat liver is intensely dependent on serum opsonin.^[^ ^104^ ^]^ In addition, mice inter‐strain variabilities have been seriously overlooked as far as the phagocytic capacity of MPS is concerned. We proposed that inter‐strain variabilities in liposome pharmacokinetic profiles could be calibrated using the corrected phagocytic rate (*K*_C_ *= K* × *(c* × *Ig)* / *(alb ×* *apo*)), especially for serum opsonin‐sensitive liposomes (e.g., FA‐functionalized liposomes), where *c*, *Ig*, *alb* and *apo* represent the total content of complement proteins, immunoglobulins, albumin, and apolipoproteins, respectively.^[^ ^105^ ^]^

### 4.3 Suppressing IgM Production

Co‐administration of immunosuppressive agents (e.g., corticosteroids, antimetabolite agents, mTOR inhibitors, anti‐CD20 antibody) or cytotoxic drugs (e.g., methotrexate, vincristine, cyclophosphamide, chlorambucil) has previously been demonstrated to diminish antibody responses to both TD and TI antigens.^[^ ^106^, ^107^ ^]^ For example, in rats administered with cyclophosphamide to deplete splenic MZB cells, the anti‐PEG IgM response was diminished.^[^ ^108^ ^]^ Therapeutic doses of Doxil also inhibit the production of anti‐PEG IgM antibodies due to cytotoxicity on B cells, which may be the reason why no Doxil related ABC phenomenon has been reported clinically. In addition, similar to the blockade of the MPS, blocking stimulation of PEG‐specific B cells in the spleen represents a strategy for lessening the production of anti‐PEG IgM antibodies. Constant and intensive antigen stimulation can block the BCR signal pathway and desensitize B cells to induce B‐cell anergy. As a result, antibody production ceased.^[^ ^51^, ^109^ ^]^ Lai et al. reported that pre‐administration of a relatively low dose (5.5 mg kg^−1^) of high molecular weight (40 kDa) free PEG led to a threefold reduction of anti‐PEG IgM antibodies compared to PBS treatment 7 days following injecting of empty PEGylated liposomes. Furthermore, the fusion of free PEG effectively saturated anti‐PEG IgM antibodies, restoring the circulation of PEGylated liposomes in mice with high titers of pre‐existing anti‐PEG IgM antibodies.^[^ ^110^ ^]^

### 4.4 Attenuating IgM Adsorption

#### 4.4.1 Structural Optimization of the PEG Moieties

Although the stealth coating using PEG greatly reduce nonspecific opsonization, prominent protein coronas have been reported to inevitably form around liposomal surfaces in the bloodstream. Antibody responses induced by protein coronas can minimize the stealth effect for repeated injections (i.e., the ABC phenomenon of PEGylated liposomes as aforementioned). Structural optimization of the PEG moieties is one of the successful approaches to evade corona formation and IgM adsorption. For instance, branched PEG‐modified liposomes induced significantly lower anti‐PEG IgM antibody levels and complement activation than linear PEG‐modified liposomes, holding promise in avoiding the ABC phenomenon, reducing CARPA, and improving the antitumor efficacy of encapsulated drugs.^[^ ^111^ ^]^ Ishida et al. reported a polyglycerol‐derived polymer lipid conjugate which was utilized as a biocompatible alternative polymer for liposome modification. They found that the hydroxymethyl pendant group in the polyglycerol repeating subunit could eliminate the production of anti‐IgM antibodies in rodents, thereby preventing the ABC phenomenon and improving the in vivo performance of polyglycerol‐coated liposomes after repeated injections.^[^ ^112^ ^]^

#### 4.4.2 Pre‐Adsorption of Functional Proteins and Antibodies

Coating with a hydrophilic polymer does prevent the adsorption of certain proteins, but paradoxically, this can also evoke recognition of liposomes by the immune system, especially with repeated administration. To overcome this issue, precoating liposomal surfaces with functional proteins and antibodies has been reported as an alternative approach to endow liposomes with stealth properties. Giulimondi et al. showed that pre‐coating of liposomes with artificial human plasma proteins corona drastically reduced the cellular recognition and uptake by circulating leukocytes in peripheral blood.^[^ ^113^ ^]^ The pre‐adsorption process has also been reported to be a versatile and convenient method for the attachment of targeting antibodies to nanocarriers’ surfaces. Compared to covalently bound antibodies that were covered by protein coronas and no longer functional, pre‐adsorbed antibodies maintained their function and were not masked by the protein coronas.^[^ ^114^ ^]^

Another prominent example is the pre‐adsorption of scFv. scFv is a single‐chain variable antibody fragment with high antigen‐binding activity and small molecular weight. Recently, our group prepared a 30‐kDa anti‐PEG scFv with a high PEG binding affinity for the amelioration of PEGylated liposome‐related ABC phenomenon. In naïve rats without anti‐PEG antibodies, pre‐deposition of such anti‐PEG scFv on the liposomal surface effectively abolished the complement activation due to the lack of Fc fragment. The anti‐PEG scFv also had negligible influence on the intrinsic performance of liposomes due to its small molecular weight. However, in liposome pre‐stimulated rats, anti‐PEG scFv pre‐adsorption effectively competed for the binding of anti‐PEG IgM antibodies and subsequently blocked complement activation, significantly ameliorating the ABC phenomenon of liposomes by a 1.5‐folds increase of the AUC values.^[^ ^115^ ^]^

Notably, we further developed an anti‐PEG scFv‐based affinity chromatography to achieve precise and efficient separation of protein coronas on PEGylated liposomes. It demonstrated a 43‐fold higher protein coronas collection efficiency than the classical centrifugation method.^[^ ^116^ ^]^ In addition, we reported a facile anti‐PEG scFv‐based separation method combined with HPLC to quantify free doxorubicin and liposomes‐encapsulated doxorubicin in plasma. The anti‐PEG scFv was used to precipitate PEGylated liposomes by simple incubation and low‐speed centrifugation (100–2000 × *g* for 10 min), demonstrating sufficient accuracy and sensitivity compared to conventional solid‐phase extraction method.^[^ ^18^ ^]^ We hope the anti‐PEG scFv could provide new effective methods for protein coronas analysis and pharmacokinetic evaluation of PEGylated liposome drugs and other PEGylated nanomedicines.

#### 4.4.3 Re‐Designing the Structure of the Targeting Ligand

Targeting ligands such as aptamers, peptides, and antibodies are anticipated to achieve a high targeting yield of liposomes to diseased tissues by recognizing corresponding receptors. For instance, a brain‐targeted peptide ligand, ^L^CDX, and its retro‐inverso peptide analog ^D^CDX, have previously been designed by our group to promote precise delivery of liposomal agents to the glioma.^[^ ^117^, ^118^, ^119^ ^]^ However, surface modification with such long (16‐amino acid), stable positively charged peptide triggered avid absorption of IgM, causing enhanced immunogenicity and rapid blood clearance. Electrostatic interaction was identified to play a crucial role because the content of IgM absorption positively correlated with the net positive charges of peptides by amino acid mutations. A short stable peptidomimetic ligand D8 with comparative targeting ability toward its receptor nAChRs has been successfully re‐designed by computer‐aided design. Compared to CDX, D8 successfully improved the immunocompatibility of brain‐targeted liposomes by attenuating nIgM absorption.^[^ ^120^ ^]^ The results provide insights into developing promising targeted liposomes by ligand structural manipulations to reduce potential immunogenicity.

### 4.5 Modulating Complement Cascade by Altering Topological Features

Complement‐mediated opsonization is a prime consequence of IgM adsorption. Therefore, modulation of the complement cascade can be a key strategy for improving the in vivo performance of liposomes. By use of proper surface chemistry design such as carboxyl, methoxyl, and amine groups is certainly a good way, but here we would like to highlight the pivotal role geometric and topological features (e.g., size and curvature) played in complement activation.

Activation of all complement pathways leads to the deposition of C3 proteolytic fragments, for example, C3b, on particle surfaces. The buried area of each surface‐bound C3b molecule is estimated to be 40 nm^2^. Due to topological constraints, most of the C3b molecules are rather difficult to deposit on the surface of particles with high curvature. Thus, small particles are deemed to effectively escape C3b opsonization, whereas they are still capable of activating the complement system and releasing anaphylactic peptides due to the assembled fluid phase C3 convertases.^[^ ^121^ ^]^ In addition, small molecule structure critically determines how immunoglobulins differentiate self from non‐self to prevent infection. Usually, the buried surface of epitope recognition by antibodies is 12–20 nm^2^. However, for large immunoglobulins such as sIgM whose cross‐sectional diameter in solution is ≈40 nm, it involves a far larger epitope‐presenting surface than a single paratope and epitope. The surface‐bound sIgM adopts a staple‐like conformation to activate complement, in which the five Fab fragments of sIgM are tightly adsorbed to the particle surface, bent about 60° relative to the Fc5 plane to expose a binding site for C1q.^[^ ^122^ ^]^ Therefore, the conformation of surface‐bound sIgM can also be greatly influenced by topological features, and so does the subsequent C1q docking. Pedersen et al.^[^ ^123^ ^]^ have explored the relationship between complement activation and the interaction between sIgM and surfaces with different curvatures. They found that in contrast to a larger or smaller size, fragments of peptidoglycan with sizes of 100 nm were more likely to trigger potent complement activation through the classical pathway. Since the as‐prepared liposomes are typically around 100 nm in diameter, their potent complement‐activating capacity can be explained. More recently, we have investigated the effects of lipid carrier morphology on FA functions and found that FA‐functionalized lipodiscs (FA‐Disc) successfully circumvented IgM‐mediated opsonization and ameliorated the in vivo performance. Owing to the distinct morphology features of FA‐Disc, FA moieties were restricted to its highly curved edges, along with the bound IgM. In this case, it eventually circumvented IgM‐mediated complement activation because the bound IgM was in an inactive conformation. The clearance of FA‐Disc as well as the IgM‐mediated ABC phenomenon was alleviated, too. FA‐Disc maintained its folate receptor binding and tumor‐targeting capacities in vivo.^[^ ^124^ ^]^ Interestingly, we also revealed that plain lipid nanodiscs displayed unique protein corona patterns. Apolipoproteins preferentially bound to the discoid surface endowed nanodiscs with distinct brain targeting capability. This work may provide new insights into morphology‐driven manipulation of the protein coronas for efficient preferential drug delivery.^[^ ^125^ ^]^

### 4.6 Manipulating IgM as a Functional Self‐Adjuvant for Humoral Immunity

The exploitation of protein coronas by recruiting functional endogenous biomolecules to the nanocarriers’ surface has emerged as a promising strategy for improving targeted drug delivery. Liposomes are surface engineered to pick up specific plasma proteins to initiate receptor‐mediated cellular recognition, internalization, and translocation across biological barriers. Potential receptors (e.g., complement receptors, immunoglobulin receptors, lipoprotein receptors, and scavenger receptors) are abundant depending on the type of plasma proteins. For example, Zhang et al.^[^ ^126^ ^]^ developed bioinspired liposomes by modifying the liposomal surface with a short peptide that specifically interacts with the lipid‐binding domain of apolipoproteins. The engineered liposomes actively absorb apolipoproteins (i.e., Apo A1, Apo E, and Apo J) in vivo, consequently exposing the receptor‐binding domain of apolipoproteins to achieve brain‐targeted delivery via multiple apolipoprotein receptor‐mediated transcytosis in mice.

IgM is the most potent immunoglobulin to activate the complement system and thus may work as an efficient immune response enhancer.^[^ ^32^ ^]^ The enhancement involves complement receptors 1 and 2 (CR1/2) and FcµR that are expressed on B cells. Since both the IgM‐CR and IgM‐FcµR pathways may contribute to humoral immunity, IgM can be utilized as a self‐adjuvant to modulate the interplay between nanovaccines and immune cells. As mentioned before, ^D^CDX‐modified liposomes heavily absorbed IgM via electrostatic interaction. It inspired us to use ^D^CDX‐modified liposomes for enhanced antigen uptake and presentation by splenic MZB cells via both CR and FcµR pathways. ^D^CDX‐modified liposomes induced a stronger and more durable IgG1 titer than plain ovalbumin‐loaded liposomes.^[^ ^127^ ^]^ Since the biological functions of IgM are diverse, this work suggested that manipulating IgM as a functional self‐adjuvant may provide new impetus for improving in vivo performance of liposome‐based nanovaccines.

## 5 Summary and Prospects

The nano‐bio interfaces are far more complex than that of conventional small molecular drugs. Although increasing clues have been learned about the in vivo performance of liposomes over the past decades and some general principles have emerged, detailed relationships of liposomes with plasma protein coronas formation, immune cells interplay, and pathophysiological response remain elusive. The theoretical system of the in vivo delivery process of liposome formulations is still in a blank state. Without this mechanistic knowledge as a guide, it is difficult to design liposomes for in vivo delivery in a controlled manner.

**Figure 4:**
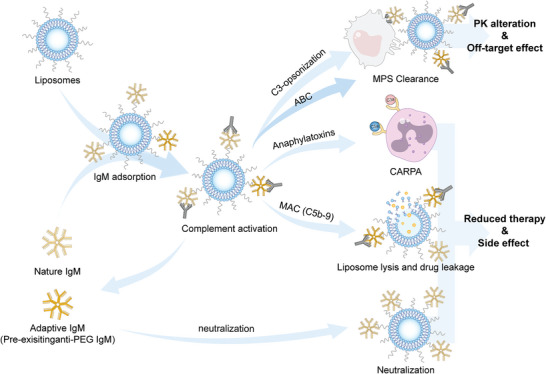
The negative regulation of IgM on physicochemical properties and in vivo biological performance of liposome‐based nanomedicine. The therapeutic efficacy of liposome‐based nanomedicine can be reduced by IgM either through altering the pharmacokinetics and “off‐target” effect, directly neutralization, or inducing side effects represented by drug leakage and CARPA. Among them, IgM‐mediated complement activation plays a crucial role in negatively regulating the in vivo biological performance of liposome‐based nanomedicine.

Among these abundant sources of protein coronas, IgM was identified to be one of the key plasma proteins regulating the in vivo performance of liposomes (especially for some targeted liposomes represented by FA‐modified liposomes). As shown in **Figure** **4**, the regulatory effect of IgM on liposomes can be generalized as follows: after injecting liposomes in blood, avid IgM (regardless of nIgM or adaptive IgM) deposits on the liposomal surface via either hydrophobic interactions, van der Waals forces, hydrogen bonds, electrostatic interaction or specific affinity. Upon binding, IgM adopts a staple‐like conformation to expose the binding sites for C1q docking, which then triggers the complement cascades via the classical pathway. Consequently, the pharmacokinetics and pharmacodynamics of liposomes are altered. On one hand, C3‐mediated opsonization exacerbates liposomal clearance by the MPS, changing the desired biodistribution. In the case of repeated administration, anti‐PEG IgM antibodies can be elicited mainly through TI pathway by splenic MZB cells, causing the ABC phenomenon. For targeted liposomes, the altered biodistribution together with shielding of the binding site by IgM leads to an “off‐target” effect. On the other, the therapeutic efficiency can be directed reduced by IgM through neutralization. In addition, the MAC (C5b‐9) formation destabilizes the liposomal membranes, causing leakage of encapsulated drug and even inducing side effects by drug burst. Anaphylatoxins released in complement activation further trigger the release of a variety of vasoactive mediators and induce CARPA.

**Figure 5:**
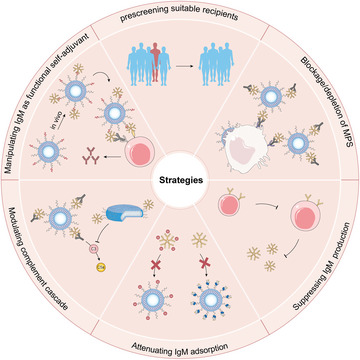
IgM‐based strategies to improve in vivo performance of liposomal nanomedicine.

Based on the regulatory effect of IgM on the delivery process in vivo, corresponding strategies can be adopted to improve the performance (**Figure** **5**).

1) Considering the individual variability of IgM, it is necessary to pre‐screen suitable recipients before medication. Adjusting doses according to the IgM levels and activities of recipients might be an alternative to calculating the dose based on body surface area. Extreme cautiousness is urged especially for patients with potentially serious side effects. Premedication with transient MPS blockade or IgM suppression might improve the therapeutic efficiency.

2) IgM adsorption and sequent complement cascade are the culprits that negatively regulate the in vivo performance of liposomes. Thus, design strategies to attenuate IgM adsorption onto liposomal surfaces such as structural optimization of the PEG moieties, pre‐adsorption of functional proteins and antibodies to shield IgM deposition, structural re‐design of the targeting ligand to weaken IgM binding affinity, modulation of complement cascade by liposomal topological features, can achieve desired surface properties and optimal in vivo performance.

3) IgM can be manipulated as a functional self‐adjuvant to modulate the interplay between liposomes and immune cells, providing new impetus for the development of liposome‐based nanovaccines for humoral immunity. Since IgM has multiple biological functions, there may be other applications to be explored based on the interaction of liposomes with IgM.

Plenty of understanding of the fundamental mechanisms governing IgM corona formation on NP surfaces is still missing, particularly in complex physiological settings rather than in simple ex vivo plasma samples. Therefore, predicting the voyage of liposomes in vivo is extremely challenging due to the knowledge gaps as well as the complexity and heterogeneity of human physiology. At least these following basic questions remain for building up the theoretical system of liposomal in vivo delivery with higher clinical translational potentials.

First, more details on the structural information of the IgM‐liposome complex are needed, especially for liposomes with specific IgM affinity like FA‐functionalized liposomes.

Second, considering different routes of administration, how do liposomes interact with IgM from other sources in addition to serum, such as gastrointestinal fluids? More principles and mechanisms need to be explored.

Third, each B cell contains 100 000–200 000 mIgM‐BCR complexes on its membrane, playing crucial roles in B cell maturation, activation, and differentiation.^[^ ^128^ ^]^ According to a recent report which determined the cryo‐electron microscopy structure of human IgM‐BCR,^[^ ^129^, ^130^ ^]^ mIgM and sIgM have similar monomeric structure, so the binding mode of liposomes with mIgM deserves further exploration for more potential biological functions.

Last but not least, we need to condense and summarize general rules from fragmented clinical information. For instance, in cancer patients, varying IgM levels and binding avidity with liposomes might be a causal factor for individual differences in response to the application of liposome‐based therapeutics. The clinical value lies in the potential of IgM as an indicator to predict the in vivo performance of liposomes, which in turn instructs the de novo design and optimization of liposomes and guides clinical individualized precision medication.

## Conflict of Interest

The authors declare no conflict of interest.

## Author Contributions

H.W., S.L., and X.W. contribute equally to this work. H.W. conducted the literature review and drafted the manuscript. S.L. and X.W. revised the manuscript and draw the figures. K.J. and H.L. provided suggestions for manuscript content. C.Z. critiqued and edited the manuscript and contributed expert advice on the topic. All authors read and approved the final manuscript.
